# Acute Acalculous Cholecystitis from Infection with Epstein–Barr Virus in a Previously Healthy Child: A Case Report

**DOI:** 10.5811/cpcem.2020.4.46301

**Published:** 2020-06-15

**Authors:** Robert Langenohl, Scott Young, Kyle Couperus

**Affiliations:** Madigan Army Medical Center, Department of Emergency Medicine, Tacoma, Washington

**Keywords:** Epstein-Barr, acalculous cholecystitis

## Abstract

**Background:**

Acute cholecystitis is the acute inflammation of the gallbladder. In adults it is most frequently caused by a gallstone(s) obstructing outflow from the cystic duct, leading to gallbladder distention and edema with eventual development of biliary stasis and bacterial overgrowth, often requiring operative management. However, in children acalculous cholecystitis is more common and is often the result of an infectious process.

**Case Report:**

Here we present a case of acute acalculous cholecystitis caused by infection with Epstein-Barr virus in an otherwise healthy three-year-old male.

**Conclusion:**

Acalculous cholecystitis is an uncommon but potentially significant complication of Epstein-Barr virus infection in the pediatric population. Emergency providers should consider this diagnosis in any child being evaluated for EBV with the complaint of abdominal pain.

## INTRODUCTION

Acute cholecystitis is described as the acute inflammation of the gallbladder. Incidence rates of gallbladder disease in children are estimated at 1.3 cases for every 1000 adult cases, although these numbers have been increasing over the past decade.^1^,^2^ Cases in adults are classically associated with gallstones obstructing outflow from the cystic duct, leading to gallbladder distention and edema with eventual development of biliary stasis and bacterial overgrowth. This is often a surgical disorder and requires operative management for definitive treatment. In children, acalculous cholecystitis, or gallbladder inflammation in the absence of gallstones, is more common, occurring in up to 70% of pediatric cases, as opposed to 5–10% of adult cases.^3^ It has several proposed mechanisms and has been shown to be related to several infectious processes. Here we present a case of acute acalculous cholecystitis caused by infection with Epstein-Barr virus (EBV) in an otherwise healthy, immunocompetent three-year-old male.

## CASE REPORT

A three-year-old male, without medical comorbidity, presented to his primary care physician’s office with progressive night-time fevers for the previous three days. His parents also stated that he had developed abdominal pain and had several non-bloody loose stools. His mother reported that his bowel movements were painful, and that he had decreased urinary output and a poor appetite. His abdominal exam at that time demonstrated diffuse tenderness without localization. Labs were ordered, but after several unsuccessful attempts were unable to be obtained. The patient was ultimately diagnosed with a viral syndrome and sent home.

Approximately 10 days later the patient was brought to a local emergency department for continued fatigue, increased “whining,” and persistent fevers. The physical exam revealed a fussy, but otherwise well-appearing male. He was alert, irritable, with slight conjunctival icterus and anterior/posterior cervical lymphadenopathy. Cardiovascular and pulmonary exams were within normal limits. His abdomen was non-distended, soft, with diffuse abdominal tenderness and he was found to have 5 centimeters (cm) hepatomegaly and 4 cm splenomegaly. Labs were notable for a leukocytosis of 63×10^3^ per microliter (/μL) (4–10×10^3^/μL); platelets of 120×10^3^/μL (150–450×10^3^/μL); and a significant elevation in serum aspartate aminotransferase (AST) and alanine aminotransferase (ALT) of 314 units per liter (U/L) (10–40 U/L) and 274 U/L (10–40 U/L), respectively. A right lower quadrant (RLQ) ultrasound was obtained to evaluate for appendicitis. The appendix was not well visualized but revealed a thickened gallbladder. A dedicated right upper quadrant (RUQ) ultrasound was then obtained showing evidence of cholecystitis with gallbladder wall thickening and edema. The patient was transferred to our tertiary care center for further management.

Upon arrival the patient was slightly tachycardic with remaining vital signs being unremarkable. The family confirmed an absence of previous medical or surgical history and denied known drug allergies. Family history was notable for a father with a history of gallstones. The patient lived in Washington state with his parents and siblings with no recent travel or camping. No sick contacts were reported. Additional testing was performed with a negative respiratory viral panel, a continued leukocytosis of 52.9×10^3^/μL (4–10×10^3^/μL), with 6% lymphocytes of which 61% were atypical. Liver function tests remained elevated with an ALT of 247 U/L (10–40 U/L) and AST of 259 U/L (10–40 U/L), a total bilirubin of 4.3 milligrams (mg) per deciliter (mg/dL) (0.3–1.0 mg/dL) with a lipase of 16 U/L (10–140 U/L). A chest radiograph was ordered, which returned with a new moderate right pleural effusion with a hazy opacity of the right hemithorax and mild contralateral shift of the mediastinum. A computed tomography (CT) of the chest/abdomen/pelvis was then obtained, which revealed a thickened gallbladder with a common bile duct not well visualized, but which appeared mildly dilated for patient’s age at 4 millimeters (mm). The CT also re-demonstrated the previously visualized large right pleural effusion and trace left pleural effusion with associated atelectasis and small volume ascites. The spleen and kidneys measured large for the patient’s age but were without abnormal appearance. There was no discrete mass or lymphadenopathy identified.

The patient was ultimately evaluated by gastroenterology, general surgery, and hematology/oncology, in the setting of significant leukocytosis for evaluation of possible leukemic process. No surgical intervention was recommended, and the patient was started on broad-spectrum antibiotics. Testing for EBV was performed in the setting of hepatosplenomegaly and leukocytosis. Immunoglobulin-M antibodies were elevated, indicating an acute infection. Symptomatic treatment was continued, and broad-spectrum antibiotics were withdrawn in the setting of an identified viral etiology. After approximately two weeks the patient was discharged from the hospital.

**CPC-EM Capsule**

What do we already know about this clinical entity?

Acalculous cholecystitis is acute gallbladder inflammation in the absence of gallstones. It is frequently seen in adults and associated with significant mortality.

What makes this presentation of disease reportable?

Acute acalculous cholecystitis in the setting of Epstein-Barr virus (EBV) infection is a rare occurrence in otherwise healthy children.

What is the major learning point?

Acute acalculous cholecystitis is an uncommon but potentially significant complication of EBV infection in the pediatric population.

How might this improve emergency medicine practice?

Emergency providers should become more aware of this process and consider this diagnosis in any child being evaluated for EBV with the complaint of abdominal pain.

## DISCUSSION

EBV belongs to the Herpesviridae family. It was first discovered in 1964 and was conclusively linked to being the causative agent of infectious mononucleosis in 1968.^4^,^5^ EBV is thought to be prevalent in the majority of the adult population with recent studies estimating that greater than 90% of the adult population are antibody positive, indicating a previous infection, thought to occur in childhood.^6^ EBV is primarily transmitted via oral secretions, although it has been reported through organ transplantation and blood transfusions.^7^ EBV initially infects epithelial cells and naïve B lymphocytes and then spreads, causing primary symptoms before it enters a latent phase when all viral proteins are no longer expressed on the cell surface. Symptoms of primary infection are generally non-specific but consist of malaise, low-grade fever, and headache. These symptoms eventually progress to include sore throat, increased fever, nausea, vomiting, and anorexia. Median symptom duration is 16 days with a gradual return to baseline, which may occur over several months.^8^

Reactivation is uncommon in the otherwise healthy patient but can cause serious, life-threatening symptoms in the immunocompromised.^9^ Treatment of EBV infection is generally symptomatic. Rarely, infection has been associated with complications including meningoencephalitis, hemolytic anemia, thrombocytopenia, myocarditis, pancreatitis, pericarditis, splenic rupture, and cholecystitis.^4^

Acute acalculous cholecystitis is defined as inflammation of the gallbladder in the absence of gallstones. It has been a known disorder for greater than 150 years but remains an elusive diagnosis.^10^ In adults it is rare and commonly associated with elderly patients who have recently undergone major surgery and tends to have a significantly elevated mortality rate.^9^ In children, however, the prognosis is generally better. Acalculous cholecystitis in the pediatric population results secondary to several mechanisms. It was previously thought to be seen only in critically ill children, or burn patients, as a result of impaired gallbladder emptying from increased use of total parenteral nutrition, increased use of opioids, and prolonged fasting.^3^ Acalculous cholecystitis has also been shown to develop in patients with autoimmune disorders such as Kawasaki disease or lupus.^11^ More recently, cases have been seen in association with infectious processes. These infections include yeasts, parasites, and several bacterial species, including Brucella, Leptospira, Salmonella, staphylococcus, and viruses such as hepatitis A, cytomegalovirus, influenza, and Epstein Barr.^12^

The diagnosis of acute acalculous cholecystitis secondary to EBV infection in the pediatric population is challenging given an unreliable, age-dependent exam. As such, it is important to have a broad differential when it comes to the febrile pediatric patient with undifferentiated abdominal pain. The most common associated risk factors include trauma, recent surgery, burns, and sepsis.^13^ Acute acalculous cholecystitis is clinically indistinguishable from classic calculous cholecystitis, and as such laboratory evaluation will have similar findings.^14^ These findings are often not specific but generally reveal a marked leukocytosis and abnormal liver function tests.^15^ Therefore, imaging is often required for diagnosis.

The appropriate imaging modality varies based on patient age, illness severity, and local protocols. Ultrasound is often the first line study, but CT may be more beneficial if the diagnosis is unclear. Ultrasound will reveal evidence of cholecystitis: gallbladder wall thickness greater than 3.5 mm, gallbladder distention, sludge, and pericholecystic fluid, in the absence of gallstones.^14^ Even in the setting of known EBV infection, imaging may be indicated. In a study by Kim et al, almost one quarter (24/94) of pediatric patients with primary EBV infection showed evidence of gallbladder abnormalities on ultrasound, specifically a thickened gallbladder wall.^14^ This suggests that gallbladder disease in the setting of an EBV infection is more common than previously thought.

Treatment options for acute acalculous cholecystitis include antibiotics, cholecystostomy, or cholecystectomy. Early studies recommend early operation for adult patients with acute acalculous cholecystitis but remains controversial in the pediatric population.^16^ More recent recommendations support a nonsurgical approach as pediatric cases are often the result of infectious processes. Broad-spectrum antibiotics are frequently initiated to cover for a possible secondary infection of enteric pathogens.^17^ In general, treatment is supportive, and patients recover over a few days.

## CONCLUSION

Acalculous cholecystitis is an uncommon but potentially significant complication of Epstein-Barr virus infection in the pediatric population. Emergency providers should consider this diagnosis in any child being evaluated for EBV with the complaint of abdominal pain. If abdominal pain or tenderness is present, it is important to consider associated biliary pathology, such as acalculous cholecystitis. Treatment is generally supportive and symptoms often resolve over several days without operative management.
